# Navigating through the PD-1/PDL-1 Landscape: A Systematic Review and Meta-Analysis of Clinical Outcomes in Hepatocellular Carcinoma and Their Influence on Immunotherapy and Tumor Microenvironment

**DOI:** 10.3390/ijms24076495

**Published:** 2023-03-30

**Authors:** Muhammad Joan Ailia, Jeong Heo, So Young Yoo

**Affiliations:** 1 BIO-IT Foundry Technology Institute, Pusan National University, Busan 46241, Republic of Korea; 2 Department of Internal Medicine, College of Medicine, Pusan National University and Biomedical Research Institute, Pusan National University Hospital, Busan 49241, Republic of Korea

**Keywords:** hepatocellular carcinoma (HCC), immunotherapy, immune checkpoint inhibitors (ICIs), programmed cell death protein 1 (PD-1), programmed cell death-ligand 1 (PDL-1), tumor microenvironment (TME)

## Abstract

This systematic review aimed to assess the prognostic significance of programmed cell death-ligand 1 (PDL-1) and programmed cell death protein 1 (PD-1) in hepatocellular carcinoma (HCC). Medline, EMBASE, and Cochrane Library database searches were conducted, revealing nine relevant cohort studies (seven PDL-1 and three PD-1). Our meta-analysis showed that PD-1/PDL-1 was a marker of poor survival, regardless of the assessment method (PD-1 overall survival (OS): hazard ratio (HR) 2.40; 95% confidence interval (CI), 1.30–4.42; disease-free survival (DFS): HR 2.12; 95% CI, 1.45–3.10; PDL-1: OS: HR 3.61; 95% CI, 2.75–4.75; and DFS: HR 2.74; 95% CI, 2.09–3.59). Additionally, high level of PD-1/PDL-1 expression was associated with aging, multiple tumors, high alpha-fetoprotein levels, and advanced Barcelona Clinic Liver Cancer stage. This high level significantly predicted a poor prognosis for HCC, suggesting that anti-PD-1 therapy is plausible for patients with HCC. Furthermore, HIF-1 induces PD-1 expression, and PD1^low^SOCS3^high^ is associated with a better prognosis. Taken together, combination therapy may be the key to effective immunotherapy. Thus, exploring other markers, such as HIF-1 and SOCS3, along with PD-1/PDL-1 immunotherapy, may lead to improved outcomes.

## 1. Introduction

Hepatocellular carcinoma (HCC) accounts for 85–90% of all primary liver cancers, with hepatitis B (HBV) or C (HCV) infection, alcohol consumption, and metabolic syndrome being the main risk factors [1]. Although various therapies are available for HCC treatment, such as local ablation, surgical resection, liver transplantation, chemotherapy, and oncolytic virotherapy, the biggest issues associated with HCC treatment are recurrence, metastasis, and frequent medication resistance [2,3,4]. The poor prognosis for HCC is primarily attributable to late-stage diagnosis, which has limited effective therapeutic options. HCC may be considered a paradigm for inflammation-induced malignancies because chronic inflammation generates a stromal environment that promotes hepatocyte changes and an immunosuppressive milieu that promotes liver cancer progression [5]. Immunotherapy is a promising treatment option for this type of cancer. Recently, immune checkpoint inhibitors (ICIs) have been shown to be highly effective in the treatment of lung, breast, bladder, and non-small cell lung cancers [6,7,8]. Programmed death protein 1 (PD-1), a glycoprotein receptor on the cell surface, is generally expressed in activated T cells, B cells, and natural killer (NK) cells. The primary PD-1 ligand, programmed death-ligand 1 (PDL-1), binds to PD-1 and inhibits antitumor immunity by triggering T-cell death and depletion [9,10].

**Figure 1:**
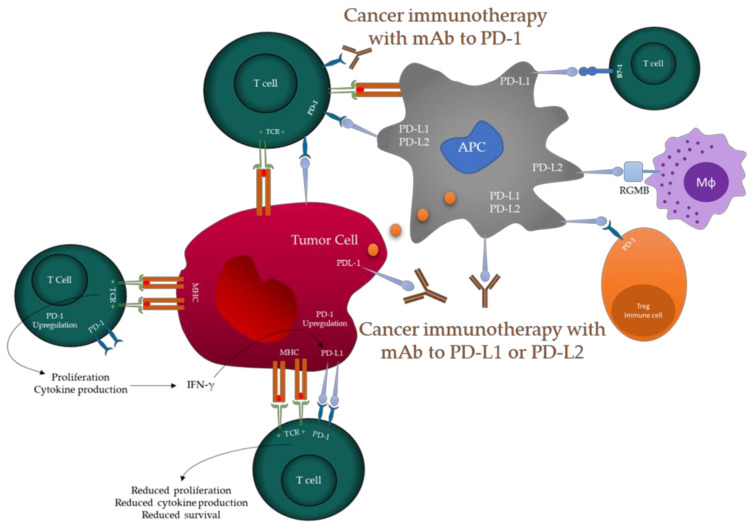
Schematic diagram for anti-programmed death protein 1 (PD-1) and anti-programmed death-ligand 1, 2 (PDL-1, PDL-2)-mediated immunotherapy for HCC. Antigen-presenting cells (APCs) acquire antigen (Ag) produced by cancer cells and deliver it to T cells to activate T-cell receptors (TCRs). Moreover, cancer cells may deliver Ag to activated T cells within the framework of MHC. During T-cell activation, PD-1 receptors are expressed on T cells and suppress immunological responses via the interaction with PD-L1 and PD-L2 on APCs and PD-L1 on cancer cells, consequently blocking the PD-1/PD-L1/PD-L2 pathway using a monoclonal antibody (mAb). Modified from [20].

PD-1/PDL-1 has been identified as a viable target for the development of potent anticancer therapies for HCC [11] and in models for comprehending the various physiological roles of inhibitory receptors. Signals from the PD1 pathway help regulate T-cell activation, T-cell destiny and activities, T-cell tolerance, and the return to immunological homeostasis [12,13] (Figure 1). However, high and sustained expression of PD1 and its ligands is ubiquitous during chronic infections and cancer. Thus, blocking the PD1 pathway can improve T-cell activity and lower the viral load and tumor burden [14,15]. The use of monoclonal antibodies to block PDL-1/PD-1 interaction represents a watershed moment in anticancer immunotherapy [16]. Furthermore, the United States Food and Drug Administration (FDA) approved sorafenib (Nexavar) and nivolumab (OPDIVO, Bristol-Myers Squibb Co.) as the first anti-PDL-1/PD-1 antibodies for the treatment of HCC in 2007 and 2017, respectively, confirming that ICIs may play a key role in HCC treatment and offer hope for patients with cancer [17,18].

However, the specific mechanism and characteristics of PD-1/PDL-1 expression, its relationship with prognosis, and the role of the tumor microenvironment (TME) and ICI in immunotherapy have not been well explored in HCC. Therefore, although PD-1/PDL-1 antibodies show promising outcomes in cancer treatment, only a small proportion of patients respond to treatment [19]. Thus, this is the first systematic review and meta-analysis undertaken to better understand the mechanisms and pathways concerning the involvement of PD-1/PDL-1 in HCC immunotherapy, ICI, and TME.

## 2. Materials and Methods

### 2.1. Search Strategy

This study was conducted in accordance with the preferred reporting criteria for systematic reviews and meta-analyses (PRISMA) and registered with PROSPERO (ID: CRD42023327021). Four major electronic databases, namely, MEDLINE, EMBASE, Web of Science, and the Cochrane Library, were searched for relevant English-language papers published through to May 2022. The electronic search was followed by a manual search of references through cross-referencing key papers. The database of the retrieved materials was managed using EndNote X20 (Thomson Reuters, New York, NY, USA). Clinical trials regarding PDL-1 and PD-1 were hand-searched on the using the database of clinicaltrails.gov, date 31st May 2022. The keywords used were “Hepatocellular carcinoma”, “Programmed death protien 1”, “Programmed death ligand 1”, “Immune check point inhibitors”, “Monoclonal antibodies”.

### 2.2. Criteria for Inclusion and Exclusion

Studies were included if all of the following six eligibility criteria were met: (1) they addressed the association of PD-1/PDL-1 in HCC; (2) they included sufficient information concerning PD-1/PDL-1; (3) clinicopathological parameters were discussed; (4) they showed an association between anti-PD-1/PDL-1, cancer progression, TME, and immune checkpoints; (5) they were written in English; and (6) appropriate consent had been obtained from the patients.

Studies were excluded if they met any of the following three exclusion criteria: (1) they comprised duplicate studies, reviews, case reports, letters, or conference proceedings; (2) the PD-1/PDL-1 association was not clearly described; or (3) they lacked sufficient data.

### 2.3. Data Extraction and Assessment of Study Quality

The data were independently extracted by two authors (MJA and SYY). Disagreements were resolved through consensus during the process. Meta-data were extracted from all studies in terms of nine categories: reference, year, country, patients (number), sample source, assessment method, cut-off, outcome, and follow-up. Clinical trial data were systematically collected and segregated based on phases I, II, and III.

### 2.4. Statistical Analysis

The Review Manager software (version 5.4; Cochrane Collaboration, Copenhagen, Denmark) was used for the statistical analysis. The association between PD-1/PDL-1 and survival were assessed using pooled HRs with 95% CIs (>1 HR value indicated low survival and vice versa). The Mantel–Haenszel pooled OR with 95% CI and the combined effective value was used to determine the association between PD-1/PDL-1 and various clinicopathological markers. An *I*^2^ score <50% suggested that there was no heterogeneity among the studies.

## 3. Results

### 3.1. Eligible Studies

**Figure 2:**
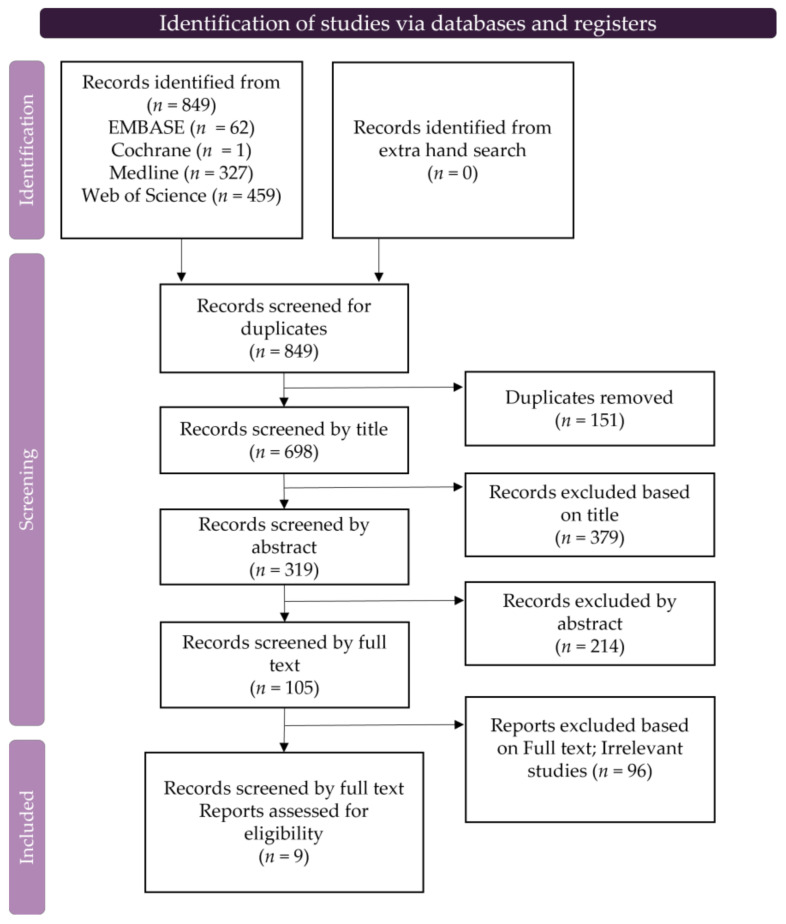
PRISMA flow chart of the search and selection procedure.

The retrieval approach described in the Methods section (Section 2) initially yielded 849 studies, of which 151 were excluded based on duplicates and 593 were excluded based on their titles and abstracts. After reviewing the full texts of the remaining articles, an additional 96 were excluded owing to missing, irrelevant, irretrievable, or duplicate data, leaving nine studies for inclusion. Studies were selected in accordance with the PRISMA flowchart (Figure 2).

### 3.2. Characteristics of the Studies Included

Nine studies were selected for final analysis. These studies were conducted in three countries and published between 2011 and 2021 (Table 1). Six studies utilized the IHC score method with a mean cut-off score > 3. Of these, two utilized ELISA, and one used flow cytometry. The total number of patients was 1284, ranging from 42 to 285, and the diagnoses included tumor stages I–IV (Table 1).

### 3.3. Survival Analysis of PD-1 and PDL-1

**Figure 3:**
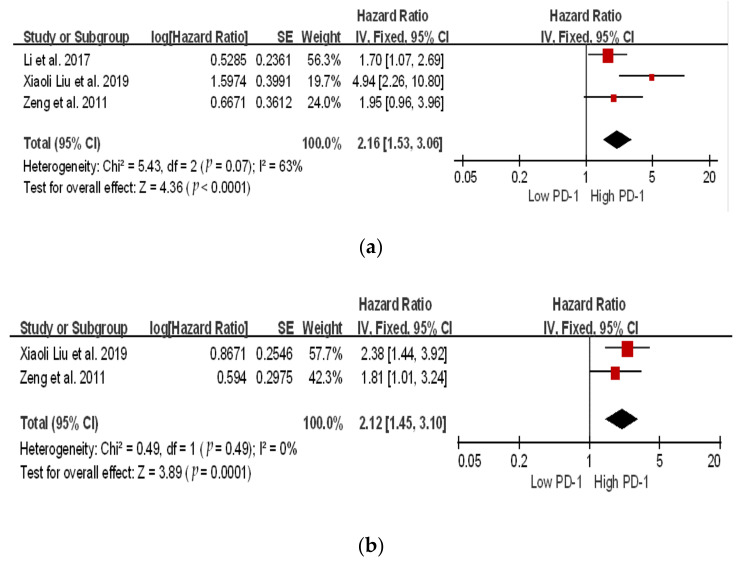
Subgroup hazard ratios assessing PD-1 expression. (**a**) Overall survival and (**b**) disease-free survival in patients with hepatocellular cancer [26,27,29]. 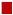 The location of the square shows the risk ratio, while the size of the square represents the individual effect of research; 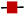 the black line depicts the study’s confidence interval at 95%; 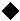 the edge of the diamond denotes the 95% confidence interval for the pooled hazard ratio.

**Figure 4:**
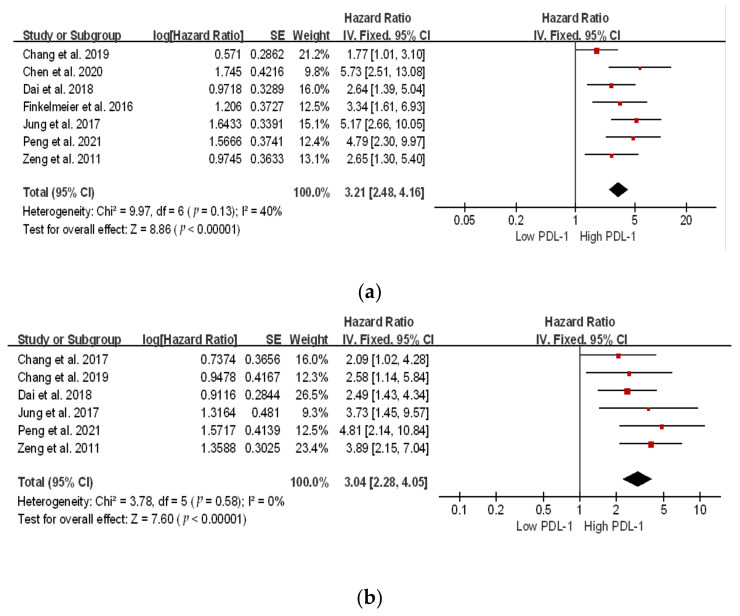
Subgroup hazard ratios assessing PDL-1 expression. (**a**) Overall survival, and (**b**) disease-free survival in patients with hepatocellular cancer [21,22,23,24,25,26,28,30]. 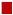 The location of the square shows the risk ratio, while the size of the square represents the individual effect of research; 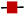 the black line depicts the study’s confidence interval at 95%; 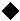 the edge of the diamond denotes the 95% confidence interval for the pooled hazard ratio.

Based on these nine studies, we evaluated the correlation between PD-1 and PDL-1 expression in 1284 patients. We identified seven studies related to PDL-1 and three related to PD-1 expression. Meta-analysis revealed a significant correlation between high and low PD-1/PDL-1 expression in both the overall survival (OS) and disease-free survival (DFS) for PD-1 (OS: hazard ratio (HR) 2.40; 95% confidence interval (CI), 1.30–4.42, *p* = 0.005; DFS: HR 2.12; 95% CI, 1.45–3.10; *p* = 0.0001), and for PDL-1 (OS: HR 3.61; 95% CI, 2.75–4.75; *p* ≤ 0.00001; DFS: HR 2.74; 95% CI, 2.09–3.59; *p* < 0.00001) (Figure 3 and Figure 4). Patients with high PD-1/PDL-1 expression showed poorer prognoses compared to their counterparts. High heterogeneity was observed for PD-1 OS (*I*^2^ = 63%); whereas, for DFS, the heterogeneity was *I*^2^ = 0%. However, for PDL-1, both the OS and DFS showed low heterogeneity (*I*^2^ = 40% and *I*^2^ = 0%, respectively).

### 3.4. Correlations between PD-1/PDL-1 and Clinicopathological Parameters

The correlations between PD-1 expression and age, sex, tumor size, and tumor number showed less heterogeneity (*I*^2^ ≤ 50%), while the correlations between PDL-1 expression and age, sex, tumor size, alpha-fetoprotein (AFP), tumor multiplicity, HBV history, and TNM stage showed less heterogeneity among the studies (*I*^2^ ≤ 50%). On the other hand, the meta-analysis results showed that, apart from AFP (OR 4.63; 95% CI, 1.13–19.09; *p* = 0.03) and tumor number (OR 1.89; 95% CI, 1.08–3.33; *p* = 0.003) in PD-1 and age (OR 1.95, 95% CI:1.24, 3.06, *p* = 0.004), tumor size (OR 2.08; 95% CI, 1.46–2.98, *p* < 0.0001) in PDL-1, the correlations between PD-1/PDL-1 expression and clinicopathological features were not statistically significant (*p* > 0.05). The relationship between high PD-1/PDL-1 levels and the clinicopathological features is shown in Table 2 and Table 3.

### 3.5. PD-1/PDL-1 and Other Markers

Co-overexpression of PDL-1 and hypoxia-inducible factor 1-alpha (HIF-1α) was found to be an independent prognostic factor for OS and DFS, and patients with high expression of both PDL-1 and HIF-1α had the worst prognosis compared with others. PDL-1 and HIF-1α exhibited high expression rates in HCC tissue at 41.11% (37/90) and 43.33% (43/90), respectively [22]. PDL-1 and SOCS3 were independent prognostic factors for OS, and patients with high PDL-1 expression (HR 5.275; 95% CI, 2.506–13.082; *p* < 0.001) and low SOCS3 expression (HR 0.210; 95% CI 0.093–0.475; *p* < 0.001) had a significantly poor prognosis.

## 4. Discussion

This systematic review and meta-analysis compared the expression levels of PD-1/PDL-1 in 1284 patients across nine retrospective cohort studies, comprising six studies concerning PDL-1 and three studies concerning PD-1. Patients with high PD-1/PDL-1 expression had poor survival and aggressive clinicopathological characteristics. We also attempted to understand the TME of HCC and collected information to address reoccurrence issues. To our knowledge, this is the first comprehensive systematic review and meta-analysis evaluating the correlation between PD-1/PDL-1 and HCC.

### 4.1. PD-1/PDL-1 Survival Assessment

Relevant studies on PD-1/PDL-1 expression in HCC were identified. The low inclusion rate (1.06%) was due to the rigorous process used to identify all studies on PD-1/PDL-1 expression in HCC. High expression of both PD-1 and PDL-1 resulted in a poor prognosis. A similar scenario has been reported in other cancers, such as breast cancer, cervical cancer, gastric cancer, nasopharyngeal carcinoma, non-small cell lung carcinoma, and renal cell carcinoma [31,32,33,34,35,36]. Furthermore, clinicopathological analysis showed that only AFP and tumor number were significantly associated with PD-1 expression, whereas PDL-1 expression was significantly correlated with age and tumor size. All other parameters may also be related to PD-1/PDL-1 expression, however, a larger cohort is required to explore this.

### 4.2. PD-1/PDL-1 and Tumor Microenvironment

**Figure 5:**
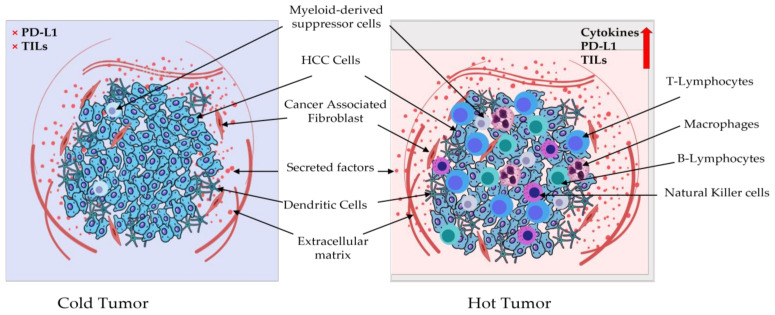
Schematic diagram representing the tumor microenvironment in hepatocellular carcinoma hot vs. cold tumor. Hot (T-cell-inflamed) tumors have significant immunological activity and significant T-cell infiltration, whereas cold (non-T-cell-inflamed) tumors are devoid of inflammation and T cells. A high expression of PDL-1, TILs and cytokines is typically associated with a hot tumor. They contain more CD8+ lymphocytes and tumor-associated macrophages (TAMs) than myeloid-derived suppressor cells (MDSCs) and cancer-associated fibroblasts (CAFs). In contrast, the TME from cold tumors is linked with low CD8+ and elevated CAFs.

The density and diversity of tumor-infiltrating immune cells are linked to prognosis and therapy efficacy prediction. Understanding the differences in immune cell composition between primary and metastatic TME may thus be a crucial element influencing the responsiveness to various immunotherapy methods [37,38]. Furthermore, immune cell composition within the TME varies substantially amongst individuals with the same cancer type, indicating that mapping the composition of immunological infiltrates and their functional status within the TME is important for both diagnosis and treatment strategy design [39]. The TME can be classified as cold (non-T-cell inflamed) or hot (T-cell inflamed), depending on the amounts of proinflammatory cytokine production and T-cell infiltration [14,40]. Hot tumors have high levels of therapeutic tumor-infiltrating lymphocytes (TILs) and cytokines, as well as high PDL-1 expression, whereas cold tumors have almost no PDL-1 expression and no T-cell infiltration [41]. In general, hot tumors respond better to immunotherapy, such as anti-programmed death-ligand (PD-L)1/PD-1 therapy [42]. The TME of HCC contains HCC-associated antigen-presenting cells (APCs), regulatory T cells (Tregs), natural killer T cells (NKTs), myeloid-derived suppressor cells (MDSCs), tumor-associated macrophages (TAMs), and TILs (Figure 5) [43].

**Figure 6:**
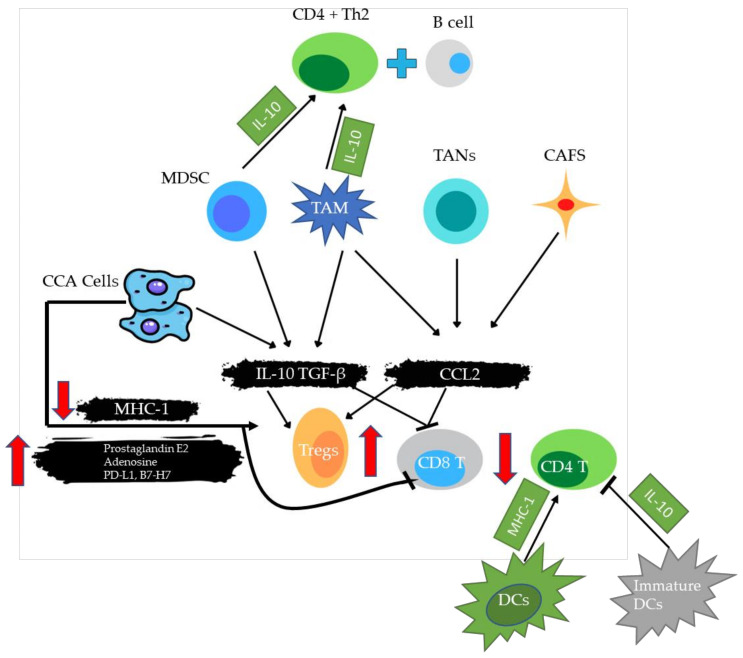
An illustration depicting the interaction between TILs and immune/cancer cells in the tumor microenvironment. Cancer cells, TAMs, and MDSCs release IL-10 and TGF-, while TAMs, TANs, and CAFs release CCL2, which recruits and boosts Tregs, while limiting CD8+ T-cell activity inside the tumor bed. Cancer cells can also directly affect the immune response through overproducing prostaglandin E2, adenosine, PD-L1, or B7-H7, or through lowering MHC-I surface expression. MSDCs and TAMs release IL10, which induces a CD4+ Th2 response with B-cell activation, both of which are effective cancer immunosurveillance mechanisms. Mature DCs stimulate CD4+ T-cell activity by increasing MHC 1 expression, whereas immature DCs decrease CD4+ T-cell activity through secreting IL-10. B7-H7, B7 homolog 7; CAFs, cancer-associated fibroblasts; CCL, C–C motif chemokine ligand; CD, cluster of differentiation; DCs, dendritic cells; IL, interleukin; MDSCs, myeloid-derived suppressor cells; MHC, major histocompatibility complex; PD-L1, programmed death-ligand 1; TANs, tumor-associated neutrophils; TAMs, tumor-associated macrophages; TGF, transforming growth factor; Tregs, regulatory T cells (modified from [53]).

TILs are crucial components of immune cells in the TME, as they participate in tumorigenesis and tumor progression, and play a key role in anti-tumor immune therapy [44]. High levels of PD-1 expression were found in liver TILs in HBV-related HCC, with this expression being correlated with portal vein tumor thrombosis, suggesting that PD-1 expression in liver tissues may be a useful prognostic marker for HBV-related HCC (Figure 6). The correlation between a high number of PD-1+ TILs and a high number of both CD4+ and CD8+ TILs suggests that PD-1+ TILs can reflect the existence of an endogenous host immune response to tumors, which is a specific pre-existing TME immune phenotype [45]. Similarly, it has been reported that a combination of a lack of PDL-1 expression and CD8+ TIL density predicts favorable survival in patients with stage III non-small cell lung cancer [40]. Gabrielson et al. reported the cumulative role of intratumoral CD3+ and CD8+ T cells and PDL-1 as prognostic markers for HCC [46]. An indirect comparison of patients with advanced squamous non-small cell lung cancer showed that, for patients with low/negative PDL-1 expression, pembrolizumab had superior OS (HR 0.43, 95% CI 0.24–0.76; *p* < 0.01/HR 0.74, 95% CI 0.40–1.38; *p* = 0.35) and better progression-free survival (HR 0.80, 95% CI 0.51–1.26; *p* = 0.33/HR 0.46, 95% CI 0.28–0.75; *p* < 0.01) than atezolizumab [47].

The expression of PD-1 on T cells can be induced through the upregulation of PDL-1 on tumor cells, as well as by other molecules. Some studies have demonstrated that PDL-1-positive tumor cells have notable immune cell infiltration in HCC, including CD3+ TILs (representing total T cells), CD8+ TILs (representing cytotoxic T cells), and TAMs (Figure 6) [29,48,49]. These findings suggest that adaptive immune resistance mechanisms may play a role. Several other studies have reported that PD-1 expression is linked to T-cell exhaustion. Thus inhibiting the PD-1 pathway could reverse this phenotype and restore antitumor immunity [50,51,52].

### 4.3. PD-1/PDL-1 Immune Check Point Inhibitors

Blocking PD-1 has been shown to have strong antitumor effects against melanoma and Hodgkin’s lymphoma, and the use of anti-PD-1 antibodies in immunotherapy has been approved by the United States FDA [54]. Monoclonal antibodies (mAbs), also known as checkpoint inhibitors (ICIs), are a class of drugs that inhibit the interaction of PD-1 and PD-L1, and thereby address the disadvantages associated with conventional anticancer therapy. Hundreds of clinical trials for anti-PD-1 and PD-L1 ICI are now ongoing. Some of them have progressed to phase III clinical trials and are benefiting a large pool of patients. We hand searched the clinical trials associated with HCC ICI [20]. Various clinical trials have targeted immune checkpoint inhibitors in HCC (Table 4, Table S1 and Table S2).

### 4.4. Combination Therapy

#### 4.4.1. Hypoxia-Inducible Factors

**Figure 7:**
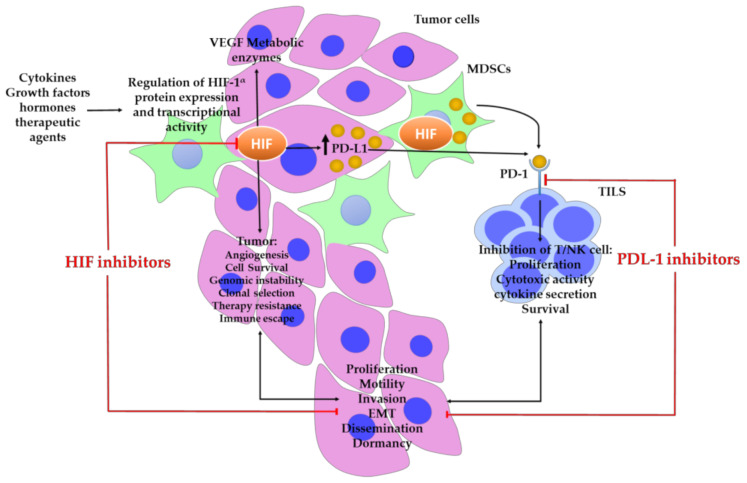
The majority of solid tumors acquire hypoxia as a result of disorganized vascularization, which deprives the tumor of optimum oxygen supply and results in increased cellular proliferation and metabolic rate. Hypoxia activates and stabilizes essential transcription factors, the hypoxia-inducible transcription factors (HIFs). HIF-1α and HIF-2α regulate the expression of several genes involved in cancer. HIFs enhance tumor growth by increasing angiogenesis, immunosuppression, EMT, and metabolic reprogramming, as well as by promoting malignant cell survival, motility, proliferation, and plasticity, and by enhancing treatment resistance and escape from a nutrient-deprived environment. Hypoxia also upregulates PD-L1 expression in cancer cells and MDSCs via HIF-1α and HIF-2α. By attaching to its receptor (PD-1), PD-L1 functions as a pro-tumorigenic factor that develops immunological tolerance within the tumor microenvironment, and suppresses antitumor immune responses by inhibiting the activity of tumor-specific TILs. Together, HIF-1α and PDL-1 inhibition decrease tumor formation and progression. More research is required to identify how additional variables in the tumor microenvironment, including cytokines, chemokines, growth factors, hormones, and therapeutic drugs, may impede the antitumor efficacy of HIF-1α inhibitors and anti-PDL-1 therapy in combination.

Hypoxia is a common characteristic of many solid cancers, including HCC [55]. Recently, it has been reported that hypoxia contributes to immune escape in cancer via a multifaceted mechanism [56]. Among them, hypoxia-inducible factors (HIFs) are major role players which are composed of an O_2_-regulated HIF-1α, HIF-2α, or HIF-3α subunits, and constitutively expressed HIF-1β subunit. HIF-α subunits undergo O2-dependent prolyl hydroxylation, which leads to protein breakdown and O_2_-dependent asparaginyl hydroxylation, which inhibits coactivator recruitment [57]. HIF-1α is a major transcription factor involved in the hypoxic response of cancer cells and activates hundreds of genes that play vital roles in angiogenesis, proliferation, glucose metabolism, invasion, and metastasis, and in resistance to radiation and chemotherapy in HCC [58,59,60,61,62]. HIF-1α positively regulates PD-L1 levels, suggesting that HIF-1α and hypoxia-induced elevation of PD-L1 expression comprise a mechanism of immune evasion by tumor cells [20,63]. HIF-1α binds directly to the transcriptionally active hypoxia response region in the PD-L1 proximal promoter, hence activating the production of PD-L1. It has also been shown that HIF-1α upregulates PDL-1 expression on MDSCs and tumor cells, contributing to cancer immune evasion. Under hypoxic conditions, inhibiting PD-L1 improves MDSC-mediated T-cell activation, accompanied by decreased IL-6 and IL-10 expression in MDSCs [64]. A combinational therapy with HIF-1α inhibitors in conjunction with PDL-1 blockade may be beneficial for patients with HCC and co-overexpression (Figure 7) [22].

#### 4.4.2. Suppressor of Cytokine Signaling 3 (SOCS3)

In addition, patients with HCC with low PDL-1 and high SOCS3 expression had a better prognosis based on their pT stage (*p* < 0.05). The co-expression of low PDL-1 and high SOCS3 may be a superior independent prognostic marker for patients with HCC. For example, Dai et al. showed that SOCS3 was useful for the diagnosis, staging, histological subtyping, prognosis, and therapeutic response in several types of cancer in the context of immuno-oncology via multiple mechanisms, and that genetic or epigenetic alterations in SOCS3 frequently predict a poor prognosis. SOCS3 expression and changes in the tumor-cell immune infiltration were predicted through the presence of immunosuppressive cells (MDSCs, CAFs, M2-TAMS, and Tregs) that promote T-cell exclusion [65]. Therefore, frequent follow-up of more patients co-expressing PDL-1 and SOCS3 could provide future insights. More studies are needed to determine whether a combination treatment of a SOCS3 stimulator and a PDL-1 blocker are advantageous for patients with HCC [25].

In Summary, immunotherapy has become popular in recent years, and identifying precise histological and genetic indicators of response to ICIs is now challenging in a variety of tumor forms, including HCC [66]. There have been a lot of novel systemic treatments for HCC utilizing ICI, but only a few subset of HCC patients appears to benefit from immunotherapy, underlining the need for a better knowledge of response factors [67]. Efforts to identify the predictive biomarkers, ICI inhibitor efficacy, and survival analysis are continuously being made [67,68,69]. To address this paradigm shift of HCC treatment, the overall picture of the PD-1/PDL-1 landscape was assessed. Our findings revealed that both PD-1 and PDL-1 are poor prognostic factors for HCC patient survival, with insights about the composition of the TME (cold vs. hot tumors), ongoing clinical trials for HCC immunotherapy utilizing PD-1/PDL-1 inhibitors, and possible combination therapies.

To our knowledge, this is the first systematic review and meta-analysis exploring the prognostic effects of PD-1/PDL-1 in HCC. While aiming to present the current landscape and future insights in relation to this matter, this study had some limitations. First, only studies published in English were searched. Second, the number of relevant studies identified was limited, with only three studies concerning PD-1 and only seven studies concerning PDL-1; thus, there may have been publication bias. Third, all the studies were conducted in East Asian countries. Consequently, we were unable to provide a global cohort. Despite these limitations, this study provides the first step in exploring PD-1/PDL-1 in combination with other markers, such as SOSC3 and HIF-1α. We consider that combination therapy is likely to be critical for immunotherapy in treating HCC, and that future research involving larger cohorts is needed to investigate this further.

## 5. Conclusions

We concluded that high PD-1/PDL-1 expression was a marker of a poor prognosis in HCC. PD-1 expression in the TILs of liver tissues may be a useful prognostic marker for HBV-related HCC. Various anti-PD-1/PDL-1 therapies are available. Exploring them in conjunction with other markers, such as SOCS3 and HIF-1α, may lead to improved HCC-related outcomes. Further studies are required to explore these aspects.

## Figures and Tables

**Table 1:** Main characteristics of studies included in the meta-analysis.

Reference	Year	Country	Patients (n)	Sample Source	Assessment Method	Cut-Off	Outcome	Follow-Up (Months)
Peng et al. [21]	2021	China	126	Tumor tissue	IHC	≥3	OS, DFS *	84
Dai et al. [22]	2018	China	90				OS, DFS *	228
Chang et al. [23]	2017	Korea	146				DFS *	33
Jung et al. [24]	2017	Korea	85				OS, DFS *	-
Chen et al. [25]	2020	China	74	Tumor tissue	IHC	≥4	OS, DFS *	-
Zeng et al. [26]	2011	China	141	Tumor tissue	IHC	≥2	OS, DFS ^†^	
Li et al. [27]	2017	China	285	Serum	ELISA	>10 ng/mL	OS **	36
Finkelmeier et al. [28]	2016	Germany	215			>0.8 ng/mL	OS *	10
Xiaoli Liu et al. [29]	2019	China	122	PMBC	Flow cytometry	12.8% CD8+ Cells	OS, PFS **	14.75

Abbreviations: CD8, cluster of differentiation 8; DFS, disease-free survival; ELISA, enzyme-linked immunosorbent assay; IHC, immunohistochemistry; PD-1, programmed death protein 1; PDL-1, programmed death ligand-1; PMBC, peripheral blood mononuclear cell; OS, overall survival. * PDL-1 expression only, ** PD-1 expression only, ^†^ Both PD-1 and PDL-1 expressions studied.

**Table 2:** Clinicopathological characteristics and pooled ORs associated with PD-1 expression.

Characteristics	Studies (n)	Participants (n)	Pooled OR (95% CI)	*p*-Value	Heterogeneity		
					*I*^2^ (%)	*p*-Value	Model
Sex (Male vs. Female)	3	407	1.39 [0.81, 2.37]	0.23	0	0.60	Fixed
Age (<50 vs. >50)	3	417	1.12 [0.73, 1.71]	0.61	26	0.26	Fixed
BCLC Stage (A vs. B)	2	229	0.52 [0.04, 7.27]	0.62	92	0.0005	Random
HBV History (Present vs. Absent)	2	261	1.25 [0.26, 5.94]	0.68	77	0.001	Random
AFP (ng/mL) (≤25 vs. ≥25)	6	646	4.63 [1.13, 19.09]	0.03 *	86	0.001	Random
Tumor Size (≤5 vs. ≥5)	6	646	0.58 [0.29, 1.16]	0.12	0	0.57	Fixed
Tumor Number (Single vs. Multiple)	4	451	1.89 [1.08, 3.33]	0.003 *	10	0.33	Fixed

Abbreviations: AFP, alpha-fetoprotein; BCLC, Barcelona Clinic Liver Cancer; CI, confidence interval; HBV, Hepatitis B virus; OR, odds ratio. * Statistically significant data.

**Table 3:** Clinicopathological characteristics and pooled ORs associated with PDL-1 expression.

Characteristics	Studies (n)	Participants (n)	Pooled OR (95% CI)	*p*-Value	Heterogeneity		
					*I*^2^ (%)	*p*-Value	Model
Sex (Male vs. Female)	6	656	1.95 [1.24, 3.06]	0.004 *	50	0.08	Fixed
Age (<50 vs. >50)	6	656	1.21 [0.88, 1.67]	0.25	30	0.21	Fixed
BCLC Stage (A vs. B)	3	283	1.04 [0.11, 9.85]	0.97	90	<0.0001	Random
HBV History (Present vs. Absent)	5	498	1.04 [0.63, 1.70]	0.88	0	0.49	Fixed
Child Pugh Score (5 vs. 6)	2	261	0.84 [0.42, 1.67]	0.62	0	0.80	Fixed
AFP (ng/mL) (≤25 vs. ≥25)	6	646	0.85 [0.61, 1.18]	0.33	38	0.16	Fixed
Tumor Size (≤5 vs. ≥5)	6	646	2.08 [1.46, 2.98]	<0.0001 *	75	0.001	Fixed
Tumor Number (Single vs. Multiple)	4	451	1.16 [0.73, 1.86]	0.52	50	<0.11	Fixed
Tumor Stage (I + II vs. III + IV)	3	264	1.39 [0.70, 2.75]	0.35	0	0.54	Fixed
Cirrhosis (Positive vs. Negative)	3	305	0.34 [0.12, 0.95]	0.84	23	0.27	Fixed

Abbreviations: AFP, alpha-fetoprotein; BCLC, Barcelona Clinic Liver Cancer; CI, confidence interval; HBV, Hepatitis B virus; OR, odds ratio. * Statistically significant data.

**Table 4:** Phase III clinical trials of immune checkpoint inhibitors for HCC.

Clinical Setting	Regimen	Starting Date	Estimated Study Completion Date	Patients	Trial Name/Number
First-line systemic	Atezolizumab–cabozantinib	10 June 2018	1 December 2023	740	COSMIC-312/NCT03755791
	SHR-1210-apatinib	10 June 2019	June 2022	543	NA/NCT03764293
	Durvalumab–tremelimumab	11 October 2017	27 August 2024	1504	HIMALAYA/NCT03298451
	Pembrolizumab–envatinib	31 December 2018	31 December 2023	794	LEAP-002/NCT03713593
	Tislelizumab	28 December 2017	May 2022	674	RATIONLALE-301/ NCT03412773
	Sintilimab-IBI305 (anti-VEGF)	11 February 2019	December 2022	595	ORIENT-32/ NCT03794440
Adjuvant resection/ablation	Nivolumab	18 April 2018	16 December 2025	545	CheckMate-9DX/ NCT03383458
	Durvalumab–bevacizumab	29 April 2019	31 May 2024	908	EMERALD-2/ NCT03847428
	Pembrolizumab	28 May 2019	30 June 2025	950	KEYNOTE-937/ NCT03867084
	Atezolizumab–bevacizumab	31 December 2019	16 July 2027	668	IMbrave-050/ NCT04102098
Adjuvant TACE	Durvalumab or Durvalumab–bevacizumab	30 November 2018	19 August 2024	724	EMERALD-1/ NCT03778957
	Pembrolizumab–lenvatinib	22 May 2020	31 December 2029	950	LEAP-012/ NCT04246177
	Nivolumab or Nivolumab–ipilimumab	15 September 2020	29 January 2024	26	CheckMate-74W/ NCT04340193
Adjuvant TACE beads	Nivolumab	8 May 2019	June 2026	522	TACE-3/NCT04268888

## Data Availability

All data needed to support the conclusions are presented in this paper. Additional data related to this study were obtained from the authors.

## Supplementary Materials

The supporting information can be downloaded at: https://www.mdpi.com/article/10.3390/ijms24076495/s1.

## References

[1] Papatheodoridis G.V., Sypsa V., Dalekos G.N., Yurdaydin C., Van Boemmel F., Buti M., Calleja J.L., Chi H., Goulis J., Manolakopoulos S. (2020). Hepatocellular carcinoma prediction beyond year 5 of oral therapy in a large cohort of Caucasian patients with chronic hepatitis B. J. Hepatol..

[2] Villanueva A., Llovet J.M. (2011). Targeted Therapies for Hepatocellular Carcinoma. Gastroenterology.

[3] Ailia M.J., Yoo S.Y. (2022). In Vivo Oncolytic Virotherapy in Murine Models of Hepatocellular Carcinoma: A Systematic Review. Vaccines.

[4] Yoo S.Y., Badrinath N., Woo H.Y., Heo J. (2017). Oncolytic Virus-Based Immunotherapies for Hepatocellular Carcinoma. Mediat. Inflamm..

[5] Heinrich B., Czauderna C., Marquardt J.U. (2018). Immunotherapy of Hepatocellular Carcinoma. Oncol. Res. Treat..

[6] Tunger A., Kiessler M., Wehner R., Temme A., Meier F., Bachmann M., Schmitz M. (2018). Immune Monitoring of Cancer Patients Prior to and During CTLA-4 or PD-1/PD-L1 Inhibitor Treatment. Biomedicines.

[7] Fan Z.Y., Liang Y., Yang X.C., Li B., Cui L.L., Luo L., Jia Y.F., Wang Y.H., Niu H.T. (2019). A meta-analysis of the efficacy and safety of PD-1/PD-L1 immune checkpoint inhibitors as treatments for metastatic bladder cancer. Onco Targets Ther..

[8] Huang M.Y., Jiang X.M., Wang B.L., Sun Y., Lu J.J. (2021). Combination therapy with PD-1/PD-L1 blockade in non-small cell lung cancer: Strategies and mechanisms. Pharmacol. Ther..

[9] Day C.L., Kaufmann D.E., Kiepiela P., Brown J.A., Moodley E.S., Reddy S., Mackey E.W., Miller J.D., Leslie A.J., DePierres C. (2006). PD-1 expression on HIV-specific T cells is associated with T-cell exhaustion and disease progression. Nature.

[10] Taube J.M., Klein A., Brahmer J.R., Xu H.Y., Pan X.Y., Kim J.H., Chen L.P., Pardoll D.M., Topalian S.L., Anders R.A. (2014). Association of PD-1, PD-1 Ligands, and Other Features of the Tumor Immune Microenvironment with Response to Anti-PD-1 Therapy. Clin. Cancer Res..

[11] Singh V., Khurana A., Allawadhi P., Banothu A.K., Bharani K.K., Weiskirchen R. (2021). Emerging Role of PD-1/PD-L1 Inhibitors in Chronic Liver Diseases. Front. Pharmacol..

[12] Greenwald R.J., Freeman G.J., Sharpe A.H. (2005). The B7 family revisited. Annu. Rev. Immunol..

[13] Riley J.L. (2009). PD-1 signaling in primary T cells. Immunol. Rev..

[14] Pauken K.E., Wherry E.J. (2015). Overcoming T cell exhaustion in infection and cancer. Trends Immunol..

[15] Iwai Y., Terawaki S., Honjo T. (2005). PD-1 blockade inhibits hematogenous spread of poorly immunogenic tumor cells by enhanced recruitment of effector T cells. Int. Immunol..

[16] Yau T., Kang Y.K., Kim T.Y., El-Khoueiry A.B., Santoro A., Sangro B., Melero I., Kudo M., Hou M.M., Matilla A. (2020). Efficacy and Safety of Nivolumab Plus Ipilimumab in Patients with Advanced Hepatocellular Carcinoma Previously Treated With Sorafenib: The CheckMate 040 Randomized Clinical Trial. JAMA Oncol..

[17] FDA, U.S FDA Grants Accelerated Approval to Nivolumab for HCC Previously Treated with Sorafenib. https://www.fda.gov/drugs/resources-information-approved-drugs/fda-grants-accelerated-approval-nivolumab-hcc-previously-treated-sorafenib.

[18] Nexavar FDA Approval History. https://www.drugs.com/history/nexavar.html.

[19] El-Khoueiry A.B., Sangro B., Yau T., Crocenzi T.S., Kudo M., Hsu C.N., Kim T.Y., Choo S.P., Trojan J., Welling T.H. (2017). Nivolumab in patients with advanced hepatocellular carcinoma (CheckMate 040): An open-label, non-comparative, phase 1/2 dose escalation and expansion trial. Lancet.

[20] Alsaab H.O., Sau S., Alzhrani R., Tatiparti K., Bhise K., Kashaw S.K., Iyer A.K. (2017). PD-1 and PD-L1 Checkpoint Signaling Inhibition for Cancer Immunotherapy: Mechanism, Combinations, and Clinical Outcome. Front. Pharmacol..

[21] Peng J.H., Tai Y., Zhao Y.X., Luo B.J., Ou Q.J., Pan Z.Z., Zhang L., Lu Z.H. (2021). Programmed death-ligand 1 expression in the tumour stroma of colorectal liver oligometastases and its association with prognosis after liver resection. Gastroenterol. Rep..

[22] Dai X.M., Pi G.L., Yang S.L., Chen G.G., Liu L.P., Dong H.H. (2018). Association of PD-L1 and HIF-1 alpha Coexpression with Poor Prognosis in Hepatocellular Carcinoma. Transl. Oncol..

[23] Chang H., Jung W., Kim A., Kim H.K., Kim W.B., Kim J.H., Kim B.H. (2017). Expression and prognostic significance of programmed death protein 1 and programmed death ligand-1, and cytotoxic T lymphocyte-associated molecule-4 in hepatocellular carcinoma. Apmis.

[24] Jung H.I., Jeong D., Ji S., Ahn T.S., Bae S.H., Chin S., Chung J.C., Kim H.C., Lee M.S., Baek M.J. (2017). Overexpression of PD-L1 and PD-L2 Is Associated with Poor Prognosis in Patients with Hepatocellular Carcinoma. Cancer Res. Treat..

[25] Chen L.X., Huang X.X., Zhang W.Z., Liu Y., Chen B., Xiang Y., Zhang R.N., Zhang M.M., Feng J., Liu S.P. (2020). Correlation of PD-L1 and SOCS3 Co-expression with the Prognosis of Hepatocellular Carcinoma Patients. J. Cancer.

[26] Zeng Z., Shi F., Zhou L., Zhang M.N., Chen Y., Chang X.J., Lu Y.Y., Bai W.L., Qu J.H., Wang C.P. (2011). Upregulation of circulating PD-L1/PD-1 is associated with poor post-cryoablation prognosis in patients with HBV-related hepatocellular carcinoma. PLoS ONE.

[27] Li N., Zhou Z., Li F., Sang J., Han Q., Lv Y., Zhao W., Li C., Liu Z. (2017). Circulating soluble programmed death-1 levels may differentiate immune-tolerant phase from other phases and hepatocellular carcinoma from other clinical diseases in chronic hepatitis B virus infection. Oncotarget.

[28] Finkelmeier F., Canli O., Tal A., Pleli T., Trojan J., Schmidt M., Kronenberger B., Zeuzem S., Piiper A., Greten F.R. (2016). High levels of the soluble programmed death-ligand (sPD-L1) identify hepatocellular carcinoma patients with a poor prognosis. Eur. J. Cancer.

[29] Liu X., Li M., Wang X., Dang Z., Jiang Y., Wang X., Kong Y., Yang Z. (2019). PD-1(+) TIGIT(+) CD8(+) T cells are associated with pathogenesis and progression of patients with hepatitis B virus-related hepatocellular carcinoma. Cancer Immunol. Immunother..

[30] Chang B.Y., Huang T., Wei H.J., Shen L.J., Zhu D., He W.J., Chen Q.F., Zhang H.H., Li Y.J., Huang R.P. (2019). The correlation and prognostic value of serum levels of soluble programmed death protein 1 (sPD-1) and soluble programmed death-ligand 1 (sPD-L1) in patients with hepatocellular carcinoma. Cancer Immunol. Immunother..

[31] Qin T., Zeng Y.D., Qin G., Xu F., Lu J.B., Fang W.F., Xue C., Zhan J.H., Zhang X.K., Zheng Q.F. (2015). High PD-L1 expression was associated with poor prognosis in 870 Chinese patients with breast cancer. Oncotarget.

[32] Gu X.B., Dong M.L., Liu Z.Y., Mi Y., Yang J., Zhang Z.G., Liu K., Jiang L., Zhang Y., Dong S.L. (2019). Elevated PD-L1 expression predicts poor survival outcomes in patients with cervical cancer. Cancer Cell. Int..

[33] Li G.P., Wang G.L., Chi F.Q., Jia Y.Q., Wang X., Mu Q.K., Qin K.R., Zhu X.X., Pang J., Xu B.X. (2021). Higher postoperative plasma EV PD-L1 predicts poor survival in patients with gastric cancer. J. Immunother. Cancer.

[34] Zhou Y.J., Shi D.B., Miao J.J., Wu H.J., Chen J.W., Zhou X.Y., Hu D.S., Zhao C., Deng W.G., Xie C.H. (2017). PD-L1 predicts poor prognosis for nasopharyngeal carcinoma irrespective of PD-1 and EBV-DNA load. Sci. Rep..

[35] Zhao Y.J., Shi F., Zhou Q., Li Y.C., Wu J.P., Wang R.B., Song Q.K. (2020). Prognostic significance of PD-L1 in advanced non-small cell lung carcinoma. Medicine.

[36] Carlsson J., Sundqvist P., Kosuta V., Falt A., Giunchi F., Fiorentino M., Davidsson S. (2020). PD-L1 Expression is Associated With Poor Prognosis in Renal Cell Carcinoma. Appl. Immunohistochem. Mol. Morphol..

[37] Giraldo N.A., Becht E., Remark R., Damotte D., Sautes-Fridman C., Fridman W.H. (2014). The immune contexture of primary and metastatic human tumours. Curr. Opin. Immunol..

[38] Fridman W.H., Pages F., Sautes-Fridman C., Galon J. (2012). The immune contexture in human tumours: Impact on clinical outcome. Nat. Rev. Cancer.

[39] Bindea G., Mlecnik B., Tosolini M., Kirilovsky A., Waldner M., Obenauf A.C., Angell H., Fredriksen T., Lafontaine L., Berger A. (2013). Spatiotemporal Dynamics of Intratumoral Immune Cells Reveal the Immune Landscape in Human Cancer. Immunity.

[40] Duan Q.Q., Zhang H.L., Zheng J.N., Zhang L.J. (2020). Turning Cold into Hot: Firing up the Tumor Microenvironment. Trends Cancer.

[41] Galon J., Bruni D. (2019). Approaches to treat immune hot, altered and cold tumours with combination immunotherapies. Nat. Rev. Drug. Discov..

[42] Zemek R.M., De Jong E., Chin W.L., Schuster I.S., Fear V.S., Casey T.H., Forbes C., Dart S.J., Leslie C., Zaitouny A. (2019). Sensitization to immune checkpoint blockade through activation of a STAT1/NK axis in the tumor microenvironment. Sci. Transl. Med..

[43] Makarova-Rusher O.V., Medina-Echeverz J., Duffy A.G., Greten T.F. (2015). The yin and yang of evasion and immune activation in HCC. J. Hepatol..

[44] Sia D., Jiao Y., Martinez-Quetglas I., Kuchuk O., Villacorta-Martin C., de Moura M.C., Putra J., Camprecios G., Bassaganyas L., Akers N. (2017). Identification of an Immune-specific Class of Hepatocellular Carcinoma, Based on Molecular Features. Gastroenterology.

[45] Chang B.Y., Shen L.J., Wang K.F., Jin J.T., Huang T., Chen Q.F., Li W., Wu P.H. (2018). High number of PD-1 positive intratumoural lymphocytes predicts survival benefit of cytokine-induced killer cells for hepatocellular carcinoma patients. Liver Int..

[46] Gabrielson A., Wu Y., Wang H., Jiang J., Kallakury B., Gatalica Z., Reddy S., Kleiner D., Fishbein T., Johnson L. (2016). Intratumoral CD3 and CD8 T-cell Densities Associated with Relapse-Free Survival in HCC. Cancer Immunol. Res..

[47] Zhang Y.X., Zhou H.Q., Zhang L. (2018). Which is the optimal immunotherapy for advanced squamous non-small-cell lung cancer in combination with chemotherapy: Anti-PD-1 or anti-PD-L1?. J. Immunother. Cancer.

[48] Liao H.T., Chen W., Dai Y.L., Richardson J.J., Guo J.L., Yuan K.F., Zeng Y., Xie K.L. (2019). Expression of Programmed Cell Death-Ligands in Hepatocellular Carcinoma: Correlation with Immune Microenvironment and Survival Outcomes. Front. Oncol..

[49] Han X., Gu Y.K., Li S.L., Chen H., Chen M.S., Cai Q.Q., Deng H.X., Zuo M.X., Huang J.H. (2019). Pre-treatment serum levels of soluble programmed cell death-ligand 1 predict prognosis in patients with hepatitis B-related hepatocellular carcinoma. J. Cancer Res. Clin..

[50] Sakuishi K., Apetoh L., Sullivan J.M., Blazar B.R., Kuchroo V.K., Anderson A.C. (2010). Targeting Tim-3 and PD-1 pathways to reverse T cell exhaustion and restore anti-tumor immunity. J. Exp. Med..

[51] Liu J., Zhang S., Hu Y., Yang Z., Li J., Liu X., Deng L., Wang Y., Zhang X., Jiang T. (2016). Targeting PD-1 and Tim-3 Pathways to Reverse CD8 T-Cell Exhaustion and Enhance Ex Vivo T-Cell Responses to Autologous Dendritic/Tumor Vaccines. J. Immunother..

[52] Liu J.H., Liu Y.H., Meng L.Y., Liu K., Ji B. (2017). Targeting the PD-L1/DNMT1 axis in acquired resistance to sorafenib in human hepatocellular carcinoma. Oncol. Rep..

[53] Liu D., Heij L.R., Czigany Z., Dahl E., Lang S.A., Ulmer T.F., Luedde T., Neumann U.P., Bednarsch J. (2022). The role of tumor-infiltrating lymphocytes in cholangiocarcinoma. J. Exp. Clin. Canc Res..

[54] Wu X.M., Gu Z.K., Chen Y., Chen B.R., Chen W., Weng L.Q., Liu X.L. (2019). Application of PD-1 Blockade in Cancer Immunotherapy. Comput. Struct. Biotechnol. J..

[55] Bosco M.C., D’Orazi G., Del Bufalo D. (2020). Targeting hypoxia in tumor: A new promising therapeutic strategy. J. Exp. Clin. Canc Res..

[56] Barsoum I.B., Koti M., Siemens D.R., Graham C.H. (2014). Mechanisms of Hypoxia-Mediated Immune Escape in Cancer. Cancer Res..

[57] Semenza G.L. (2019). Pharmacologic Targeting of Hypoxia-Inducible Factors. Annu. Rev. Pharmacol..

[58] Semenza G.L. (2003). Targeting HIF-1 for cancer therapy. Nat. Rev. Cancer.

[59] Wu Q., You L., Nepovimova E., Heger Z., Wu W., Kuca K., Adam V. (2022). Hypoxia-inducible factors: Master regulators of hypoxic tumor immune escape. J. Hematol. Oncol..

[60] Schito L., Semenza G.L. (2016). Hypoxia-Inducible Factors: Master Regulators of Cancer Progression. Trends Cancer.

[61] Triner D., Shah Y.M. (2016). Hypoxia-inducible factors: A central link between inflammation and cancer. J. Clin. Investig..

[62] Semenza G.L. (2021). Intratumoral Hypoxia and Mechanisms of Immune Evasion Mediated by Hypoxia-Inducible Factors. Physiology.

[63] Chang Y.L., Yang C.Y., Lin M.W., Wu C.T., Yang P.C. (2016). High co-expression of PD-L1 and HIF-1alpha correlates with tumour necrosis in pulmonary pleomorphic carcinoma. Eur. J. Cancer.

[64] Noman M.Z., Desantis G., Janji B., Hasmim M., Karray S., Dessen P., Bronte V., Chouaib S. (2014). PD-L1 is a novel direct target of HIF-1α, and its blockade under hypoxia enhanced MDSC-mediated T cell activation. J. Exp. Med..

[65] Dai L.R., Tao Y.R., Shi Z.M., Liang W.L., Hu W.H., Xing Z., Zhou S.L., Guo X.Y., Fu X.D., Wang X.J. (2022). SOCS3 Acts as an Onco-immunological Biomarker with Value in Assessing the Tumor Microenvironment, Pathological Staging, Histological Subtypes, Therapeutic Effect, and Prognoses of Several Types of Cancer. Front. Oncol..

[66] Muthukutty P., Woo H.Y., Ragothaman M., Yoo S.Y. (2023). Recent Advances in Cancer Immunotherapy Delivery Modalities. Pharmaceutics.

[67] Rizzo A., Ricci A.D., Di Federico A., Frega G., Palloni A., Tavolari S., Brandi G. (2021). Predictive Biomarkers for Checkpoint Inhibitor-Based Immunotherapy in Hepatocellular Carcinoma: Where Do We Stand?. Front. Oncol..

[68] Viscardi G., Tralongo A.C., Massari F., Lambertini M., Mollica V., Rizzo A., Comito F., Di Liello R., Alfieri S., Imbimbo M. (2022). Comparative assessment of early mortality risk upon immune checkpoint inhibitors alone or in combination with other agents across solid malignancies: A systematic review and meta-analysis. Eur. J. Cancer.

[69] Rizzo A., Cusmai A., Gadaleta-Caldarola G., Palmiotti G. (2022). Which role for predictors of response to immune checkpoint inhibitors in hepatocellular carcinoma?. Expert Rev. Gastroenterol. Hepatol..

