# Supplementary material for: Navigating through the PD-1/PDL-1 Landscape: A Systematic Review and Meta-Analysis of Clinical Outcomes in Hepatocellular Carcinoma and Their Influence on Immunotherapy and Tumor Microenvironment
Source: Int J Mol Sci. 2023 Mar 30;24(7):6495. doi: 10.3390/ijms24076495 (PMC10095164; doi:10.3390/ijms24076495)
Supplement: Supplementary file 1 [file ijms-24-06495-s001.zip › ijms-2255278-supplementary.pdf]

Table S1 Phase I clinical trials of immune checkpoint inhibitors for HCC.

Regimen	Starting date	completion date	Patients	Trial name/number
FGF401, PDR001	December 29, 2014	May 30, 2019	172	NCT02325739
Vaccinia Virus Ankara Vaccine Expressing p53, Pembrolizumab	June 14, 2016	December 31, 2022	19	NCT02432963
SF1126, Nivolumab	March 27, 2017	June 29, 2020	7	NCT03059147
INCAGN01876, Nivolumab, Ipilimumab	April 25, 2017	November 9, 2021	145	NCT03126110
Nab-Rapamycin, Nivolumab	August 24, 2017	December 2, 2021	34	NCT03190174
Toripalimab	March 14, 2018	August 2023	198	NCT03474640
Cabozantinib, Durvalumab, Tremelimumab	October 22, 2018	June 30, 2023	117	NCT03539822
TQB2450, Anlotinib	April 23, 2019	January 1, 2021	60	NCT03825705
JS001	May 1, 2019	October 31, 2022	20	NCT03966209
GB1275, Pembrolizumab	August 13, 2019	April 11, 2022	61	NCT04060342
Regorafenib + Nivolumab	March 16, 2020	August 2024	78	NCT04170556
Camrelizumab	September 21, 2020	July 2023	20	NCT04564313
OC-001	October 6, 2020	November 2022	80	NCT05339321
Anlotinib hydrochloride + TQB2450	April 30, 2021	July 30, 2024	20	NCT04888546
Poly ICLC	October 1, 2021	September 30, 2024	10	NCT04260802
PF-07263689, Sasanlimab	October 20, 2021	December 6, 2022	10	NCT05061537
Ipilimumab + pembrolizumab + durvalumab	November 1, 2021	October 30, 2025	100	NCT05187338

Table S2 Phase II clinical trials of immune checkpoint inhibitors for HCC.

Regimen	Starting date	completion date	Patients	Trial number
Pembrolizumab	May 31, 2016	December 30, 2023	156	NCT02702414
Durvalumab, Tremelimumab	July 5, 2016	December 31, 2022	54	NCT02821754
Pembrolizumab	February 15, 2018	December 31, 2023	30	NCT03316872
PD-1 mAb, PolyIC	October 22, 2018	October 22, 2023	60	NCT03732547
Cabozantinib, Durvalumab, Tremelimumab	October 22, 2018	June 30, 2023	117	NCT03539822
Pembrolizumab	November 1, 2018	November 1, 2033	200	NCT03755739
N-803 + Pembrolizumab, N-803 + Nivolumab, N-803 + Atezolizumab, N-803 + Avelumab, N-803 + Durvalumab, N-803 + Pembrolizumab + PD-L1 t-haNK, N-803 + Nivolumab + PD-L1 t-haNK, N-803 + Atezolizumab + PD-L1 t-haNK, N-803 + Avelumab + PD-L1 t-haNK, N-803 + Durvalumab + PD-L1 t-haNK	December 11, 2018	December 2023	147	NCT03228667
TQB2450, Anlotinib	April 23, 2019	January 1, 2021	60	NCT03825705
IMC-001	September 9, 2019	September 26, 2026	48	NCT04196465
Sintilimab	November 16, 2019	October 31, 2022	116	NCT04167293
Cabozantinib	January 30, 2020	September 2023	46	NCT04435977
Regorafenib, Nivolumab	March 16, 2020	August 2024	78	NCT04170556
axitinib plus toripalimab	May 1, 2020	June 30, 2022	60	NCT04010071
Durvalumab	October 5, 2020	December 31, 2025	18	NCT04108481
Durvalumab	October 15, 2020	December 31, 2024	37	NCT04913480
Sintilimab	December 26, 2020	December 1, 2021	10	NCT04653389
Pembrolizumab + Regorafenib (Stivarga, BAY73-4506)	February 3, 2021	May 15, 2024	95	NCT04696055
oxaliplatin, fluorouracil, and leucovorin	July 1, 2021	December 31, 2022	49	NCT04994236
Regorafenib, Camrelizumab, Toripalimab, Pembrolizumab	October 1, 2021	December 1, 2022	20	NCT05048017
ipilimumab +pembrolizumab +durvalumab	November 1, 2021	October 30, 2025	100	NCT05187338
Cabozantinib	December 14, 2021	December 14, 2024	40	NCT04767906
pembrolizumab, Sintilimab, Camrelizumab, Duvarizumab	April 10, 2022	December 31, 2023	15	NCT05322187
Durvalumab	April 11, 2022	December 30, 2023	30	NCT04945720
Regorafenib, Toripalimab	July 25, 2022	June 30, 2024	32	NCT05485909
Envafolimab, Lenvatinib Combined With TACE	October 30, 2022	October 30, 2025	30	NCT05582109
Durvalumab, Lenvatinib	April 2023	June 2024	25	NCT05312216
